# Supplementary material for: The genome sequence of the Bog Ant Fly, Microdon myrmicae Schönrogge et al., 2002
Source: Wellcome Open Res. 2025 Mar 17;10:134. [Version 1] doi: 10.12688/wellcomeopenres.23903.1 (PMC12485359; doi:10.12688/wellcomeopenres.23903.1)
Supplement: Peer review history

# Reviewer report: Reviewer response for version 1

**DOI:** 10.21956/wellcomeopenres.26370.r121552

**Published:** 2025-05-09

**Authors:** Komal Kumar Bollepogu Raja

**Affiliations:** 1 Baylor College of Medicine, Houston, USA

The manuscript by Wolton J., Robert presents the genome assembly of *Microdon* *myrmicae* with a total length of 1,366.18 Mb and 8 chromosomal pseudomolecules. The author has identified 13,398 protein-coding genes. The background sufficiently introduces the taxonomical, biological and ecological aspects of the organism. The assembly quality metrics suggest that the genome sequence is of high quality. For instance, the BUSCO is 94.8% and the kmer completeness id 98.37%. Further, the percentage of assembly mapped to chromosome is also impressive at 98.86%. The methodology is robust and allows for reproducibility. Overall, all standard bioinformatics tools have been used for the assembly and the manuscript is well written. The genome sequence is a good resource for other researchers.

Are sufficient details of methods and materials provided to allow replication by others?

Yes

Is the rationale for creating the dataset(s) clearly described?

Yes

Are the datasets clearly presented in a useable and accessible format?

Yes

Are the protocols appropriate and is the work technically sound?

Yes

Reviewer Expertise:

Single cell genomics, Bioinformatics, genomics, and neurobiology

I confirm that I have read this submission and believe that I have an appropriate level of expertise to confirm that it is of an acceptable scientific standard.

# Reviewer report: Reviewer response for version 1

**DOI:** 10.21956/wellcomeopenres.26370.r121549

**Published:** 2025-05-05

**Authors:** Henrique Antoniolli

**Affiliations:** 1 Universidade Federal do Rio Grande do Sul (UFRGS), Porto Alegre, Brazil

The present study reports a highly contiguous genome assembly for a bog ant fly, *Microdon myrmicae*. The background section brings an interesting overview on its general biology, however does not clearly state the motivations for sequencing its genome. Protocols are well described and software are clearly referenced. The mitochondrion sequence should be deposited along with its gene annotation. The data produced yielded satisfactory results on assembly statistics, therefore I recommend indexing this paper.

Are sufficient details of methods and materials provided to allow replication by others?

Yes

Is the rationale for creating the dataset(s) clearly described?

Yes

Are the datasets clearly presented in a useable and accessible format?

Yes

Are the protocols appropriate and is the work technically sound?

Yes

Reviewer Expertise:

Genomics, evolution

I confirm that I have read this submission and believe that I have an appropriate level of expertise to confirm that it is of an acceptable scientific standard.
